# Preparation of an N–S dual-doped black fungus porous carbon matrix and its application in high-performance Li–S batteries

**DOI:** 10.3389/fchem.2023.1288013

**Published:** 2023-12-20

**Authors:** Liping Zhao, Ye Zhao, Lihe Zhao, Gang Liu

**Affiliations:** ^1^ Institute of Chemical and Industrial Bioengineering, Jilin Engineering Normal University, Changchun, China; ^2^ School-Enterprise Joint Technology Innovation Laboratory of Novel Molecular Functional Materials of Jilin Province, Changchun, China; ^3^ FAW Tooling Die Manufacturing Co., Ltd., Changchun, China; ^4^ Daqing Oilfield Design Institute Co., Ltd., Daqing, China

**Keywords:** biomass, black fungus, nitrogen–sulfur dual-doped, Li–S battery, positive electrode

## Abstract

A nitrogen–sulfur dual-doped black fungus porous carbon (NS-FPC) matrix was prepared with natural black fungus as the carbon source and cysteine as the nitrogen–sulfur source. A black fungus-based solution was obtained by hydrothermal treatment. After further carbonization activation and combination with sulfur processing, the NS-FPC/S positive electrode materials were prepared. The uniform recombination of biomass carbon provides an efficient conductive framework for sulfur. The porous structure is conducive to the transport of electrolytes. Heteroatom doping can provide a more active site. The structure and composition analyses of the materials were carried out using X-ray diffraction (XRD). The electronic binding energy and bonding state were analyzed by X-ray photoelectron spectroscopy (XPS). The morphology was observed by scanning electron microscopy and transmission electron microscopy. The specific surface area and pore size distribution were analyzed using an N_2_ adsorption–desorption experiment. Sulfur loading was determined through thermogravimetric analysis. The electrochemical performance of NS-FPC/S in Li–S batteries was systematically investigated. The result shows that the NS-FPC/S electrode maintains more than 1,000 mAh g^-1^ reversible capacity after 100 cycles at 0.2 C current density, with a capacity retention of 85%. The cycle and rate performance are both considerably superior to those of traditional activated carbon materials.

## 1 Introduction

With the development of the automotive power battery industry and the thirst for clean energy, positive electrode materials (the “heart” of Li-ion batteries) have been put forward higher requirements. Developing positive electrode materials with high energy density, high safety performance, low cost, and long cycling life has become the key to achieving large-scale applications of electric vehicles. Sulfur positive electrode materials have become one of the most promising candidate materials due to their high theoretical specific capacity, safety, and affordability. An Li–S battery refers to a type of a battery system that uses sulfur (or sulfur compounds) as the positive electrode and lithium or lithium-storage materials as the negative electrode (Fu et al., 2023; Yu et al., 2023). Compared with traditional Li-ion batteries, Li–S batteries possess higher theoretical specific capacity (1,675 mAh g^-1^) and higher theoretical energy density (2,600 Wh kg^-1^), making them the most promising secondary batteries for next-generation commercial applications (Zhao et al., 2020; Rayappan et al., 2023).

Except for high capacity, Li–S batteries are provided with some other advantages. First, mainly applying sulfur, an abundant resource, as the active substance, the overall cost has been reduced. Second, Li–S batteries are basically non-toxic, harmless, and relatively environmentally friendly. Third, the safety performance of Li–S batteries is relatively good, and the working temperature range is also relatively wide. However, there are still many problems in the actual application of Li–S batteries. The most important problems are as follows: 1) as the main positive electrode material, sulfur has poor electronic and ion conductivity, with an electronic conductivity of only 5 × 10^−30^ S cm^-1^. The insulation of sulfur leads to slow electron and ion transfer during the charging–discharging process. Poor reaction kinetics limit the utilization efficiency of active substances. 2) In the charging–discharging process, the volume effect of sulfur positive electrodes is very obvious, which is caused by the density difference between sulfur and lithium sulfide. During the entire charging–discharging process, the maximum difference in the volume between the front and rear is nearly 80%. Such a large volume expansion will cause damage to the electrode structure, thereby affecting the stability of the battery. 3) The intermediate product lithium sulfide produced will have a “shuttle effect.” This can lead to significant waste of active substances, thus affecting the capacity and cycling performance of the battery (Zhao et al., 2022a; Chen et al., 2023a).

In recent years, the electrochemical performance of Li–S batteries has been significantly improved through various modification strategies, especially compounding with the porous carbon composite method. Carbon materials have the advantages of diverse structures, rich surfaces, strong controllability, and good chemical stability, making them an ideal candidate for electrochemical energy storage materials. With the rapid development of micro–nano-carbon and its composite materials, their unique structure and excellent electrochemical performance provide new opportunities for their application in the field of electrochemical energy storage. Carbon materials have good structural stability and can accommodate large volume changes. Furthermore, carbon materials with a high specific surface area can effectively absorb more lithium sulfide and alleviate its dissolution in electrolytes. In addition, the combination of sulfur elemental and carbon materials can greatly enhance conductivity (Sun et al., 2023a; Dong et al., 2023; Meng et al., 2023).

Among those numerous carbon materials, natural biomass carbon has gradually become an ideal carbon source due to its rich sources, unique structure, easy access, rapid regeneration, low cost, and environmentally friendly nature. It has attracted the attention of a large number of researchers (Li et al., 2023a; Cui et al., 2023; Jing et al., 2023; Wu et al., 2023; Zhong et al., 2023; Zhu et al., 2023).

However, most of the reported sulfur surface loading and energy density are low, with poor cycling stability, complex preparation processes, and high preparation costs. This is because only applying pure carbon material as the conductive skeleton of sulfur results in low polarity. Polysulfide is mainly bound by physical adsorption to inhibit the occurrence of a shuttle effect, so the sulfur fixation capacity is limited. Moreover, the presence of pure carbon is mostly in the form of sp^3^ hybridization, with a low degree of graphitization and relatively poor conductivity (Li et al., 2023b; Sun et al., 2023b; Huang et al., 2023; Liang et al., 2023). Therefore, finding a simple and mild method to functionalize and modify carbon materials to further improve their conductivity and chemical adsorption capacity for polysulfide is important. It can effectively promote the redox of polysulfide in the charge–discharge process and improve the cycle life of Li–S batteries, especially the discharge capacity and cycle stability under the condition of high sulfur surface density.

Many studies have demonstrated that heteroatom-doped carbon materials can significantly improve the electrochemical performance of Li–S batteries (Zhao et al., 2019; Wang et al., 2023). This is because heteroatom doping can increase the force between the oxygen-containing functional group and the sulfur atom in the carbon material, which can prevent the dissolution of lithium polysulfide in the electrolyte so as to inhibit the shuttle effect (Dai et al., 2023; Hu et al., 2023; Jiang et al., 2023). The experimental results indicate that doping with nitrogen and phosphorus can improve the polarity of electrode materials, enhance the binding force between porous carbon matrix and elemental sulfur, and alleviate the shuttle effect (Zhao et al., 2019; Zhao et al., 2020).

In order to solve the above problems and improve the performance of sulfur positive electrodes, biomass carbon materials were introduced into the positive electrode materials of Li–S batteries in this work. Adopting black fungus as the carbon source and template and cysteine as the nitrogen–sulfur source, a nitrogen–sulfur dual-doped black fungus porous carbon/sulfur (NS-FPC/S) composite was synthesized. Black fungus is mainly composed of polysaccharides, which are mainly composed of carbon elements, so it can be used as a good carbon source. Cysteine (C_5_H_11_O_2_NS) is an amino acid containing both amino (-N_2_H) and mercapto (-SH) groups, which can be a rich source of nitrogen and sulfur. Compared to previous works (Zhao et al., 2022a; Zhao et al., 2022b), the novelty of this article lies in introducing natural black fungus as the carbon source, which reduced the experiment cost. Furthermore, adopting cysteine as a reagent to introduce both nitrogen and phosphorus heterogeneous elements simplified the experimental steps. Only adopting one reagent of cysteine, two heterogeneous elements were introduced simultaneously, which avoided the complicated and tedious doping process. This study has the advantages of simple process and low cost and focuses on green environmental protection.

## 2 Synthesis and characterization of materials

### 2.1 Reagents

Black fungus was collected from Paektu Mountain in Jilin Province of China. Cysteine, potassium hydroxide, sublimated sulfur (S_8_, 99.95%), LiTFSI (99.9%), 1,3-dioxy cyclopentane (DOL), dimethoxyethane (DME), lithium nitrate (LiNO_3_, 99.9%), and N-methylpyrrolidone (NMP) were purchased from Aladdin Biochemical Technology Co., Ltd. Hydrochloric acid and anhydrous ethanol were purchased from China National Pharmaceutical Group Chemical Reagent Co., Ltd. Deionized water was prepared in-house. An lithium metal sheet and conductive agent (super-P) were purchased from Wuxi Xinneng Lithium Industry Co., Ltd. Button-type battery cases (CR2032) were purchased from Shenzhen Weifeng Electronics Co., Ltd. Polyvinylidene fluoride (PVDF) was purchased from Solvay Fine Chemicals Co., Ltd. Aluminum foil (Al) was purchased from Beijing Institute of Nonferrous Metals. A separator (Celgard 2400) was purchased from Celgard Corporation of the United States. An lithium metal sheet (Li, 99.99%) was purchased from Tianjin Zhongneng Lithium Industry Co., Ltd. High-purity argon (Ar, 99.99%) and nitrogen (N_2_, 99.99%) were purchased from Changchun Zhongsheng Gas Co., Ltd.

### 2.2 Synthesis of materials

A measure of 6 g of dried black fungus was completely immersed in 80 mL of deionized water, transferred to 100 mL of polytetrafluoroethylene-lined autoclave, and then subjected to hydrothermal treatment at 120°C for 24 h. After cooled to room temperature, a uniform black fungus-based solution was obtained by ultrasonic treatment. Afterward, 4 g of cysteine was added to the above hydrothermal black fungus-based solution. After continuous stirring, a uniformly dispersed solution was obtained. Most of the moisture was removed using an air-drying oven, and the remaining moisture was removed through freeze-drying. Then, the sample was calcined at 800°C for 2 h in a tube furnace in an nitrogen atmosphere, at the heating rate of 5°C/min to obtain a preliminary carbon material of black fungus. Furthermore, the subsequent activation process was performed. The above products were mixed with KOH powder (the weight ratio of KOH/black fungus was 3:1) and then dried at 110°C to remove water. Then, the samples were heated at 800°C for 2 h under nitrogen flow. After activation, the sample was washed several times with 1M HCl solution and distilled water and dried at 60°C to obtain a black fungus porous carbon matrix, which was named nitrogen–sulfur dual-doped black fungus porous carbon (NS-FPC).

Then, NS-FPC and sublimated sulfur (S) were mixed (at a mass ratio of 1:3) and ground using a mortar for more than 30 min. An appropriate amount of carbon disulfide was dropped during the grinding process to increase the contact between sulfur and composite materials. After that, the ground sample was placed into a weighing bottle, and the bottle was covered with tin foil. Then, the mixed sample was placed in a vacuum oven at 155°C for 12 h for thermal diffusion. Finally, the NS-FPC/S positive electrode material was obtained. The preparation process schematic diagram of the material is shown in Supplementary Figure S1.

### 2.3 Characterization of materials

X-ray diffraction (XRD) was executed using an X'Pert Pro diffractometer manufactured in the Netherlands. The radiation source is Cu-Kα, wavelength is 1.5406 Å, and scanning angle range is 5–80°. X-ray photoelectron spectroscopy (XPS) was executed using an Axis Ultra DLD (Kratos) X-ray photoelectron spectrometer. The light source is Al-Kα, with a background vacuum of 3 × 10^−7^ Pa. Scanning electron microscopy (SEM) was executed using a Hitachi S-4700 instrument manufactured in Japan, with an acceleration voltage of 25 kV. Transmission electron microscopy (TEM) was conducted using an FEI Tecnai G2 F30 instrument, with an acceleration voltage of 300 kV. The Brunauer-Emmett-Teller (BET) test was performed and analyzed using a nitrogen/desorption instrument manufactured in the United States (model: Micromeritics ASAP 2010). The thermogravimetric (TG) test was implemented using an SDT Q600 thermogravimetric analyzer manufactured by TE Company in the United States. The atmosphere was nitrogen, with a temperature ranging from room temperature to 600°C at a heating rate of 10°C per minute.

### 2.4 Battery assembly and electrochemical performance testing

Electrode fabrication: The mass ratio of the active material, conductive agent, and binder was set at 7:2:1. A small amount of 1-methyl-2-pyrrolidone (NMP) was added to prepare a black slurry with appropriate viscosity. The adhesive was a mixture of polyvinylidene fluoride (PVDF) and NMP taken at a mass ratio of 1:10, with a dissolution temperature of 50°C. The conductive agent was acetylene black (super-P). The black slurry obtained was coated on clean aluminum sheets after 2 h of magnetic stirring. After drying at 60°C for 6 h, the slurry was put into a tablet press for pressing under a pressure of 18 MPa. After drying the completed electrode for 6 h, the final sulfur-containing positive electrode was obtained.

Battery assembly: The assembly sequence was as follows: positive electrode shell→sulfur positive electrode→separator (two layers)→electrolyte (25–30 μL)→lithium plate→spring plate→ negative electrode shell. All battery cases were of CR2032 button type. Pure metal lithium plates were used as the negative electrode, and microporous polypropylene was used as the separator (double layers). The selected electrolyte was LiN(CF_3_SO_2_) _2_/DOL + DME (volume ratio of 1:1). A 4% mass ratio of LiNO_3_ was also added.

Constant current charging–discharging test: After placing the assembled battery for 3 h, a constant current charging–discharging test was conducted using a LAND CT2001A multi-channel battery detection system. The voltage range was 1.5–3 V, and the current density was varied from 0.1 C to 3 C as needed. The testing conditions were room temperature.

Cyclic voltammetry (CV) and electrochemical impedance (EIS) test were performed using the Shanghai Chenhua electrochemical workstation (CHI660C). The scanning rate of the CV test was 0.1 mV s^-1^, and the voltage range was 1.5–3 V. The frequency range of the electrochemical impedance test was 0.01 Hz–100 kHz, and the voltage amplitude was 5 mV.

## 3 Results and discussion

### 3.1 XRD analysis of materials

**FIGURE 1:**
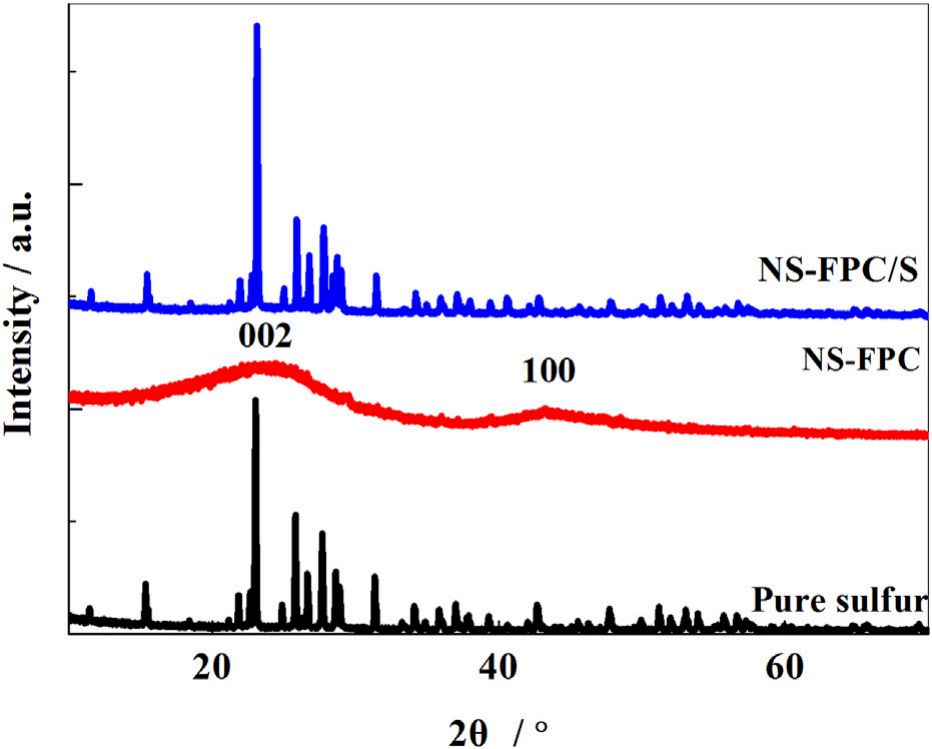
XRD patterns of sulfur, NS-FPC, and NS-FPC/S samples.

Figure 1 shows the XRD patterns of elemental sulfur, NS-FPC, and NS-FPC/S samples. It can be seen that the NS-FPC and NS-FPC/S samples exhibit two obvious peaks at 24.4° and 43.1°, which correspond to the (002) and (100) crystal planes of graphite carbon, respectively. Both the peaks are wide, indicating that the carbon structure is in the form of amorphous carbon. NS-FPC reveals only a carbon peak. On the other hand, NS-FPC/S shows no other peak except sulfur and carbon peaks, which indicates that this method of preparation results in materials of high purity.

### 3.2 XPS analysis of materials

**FIGURE 2:**
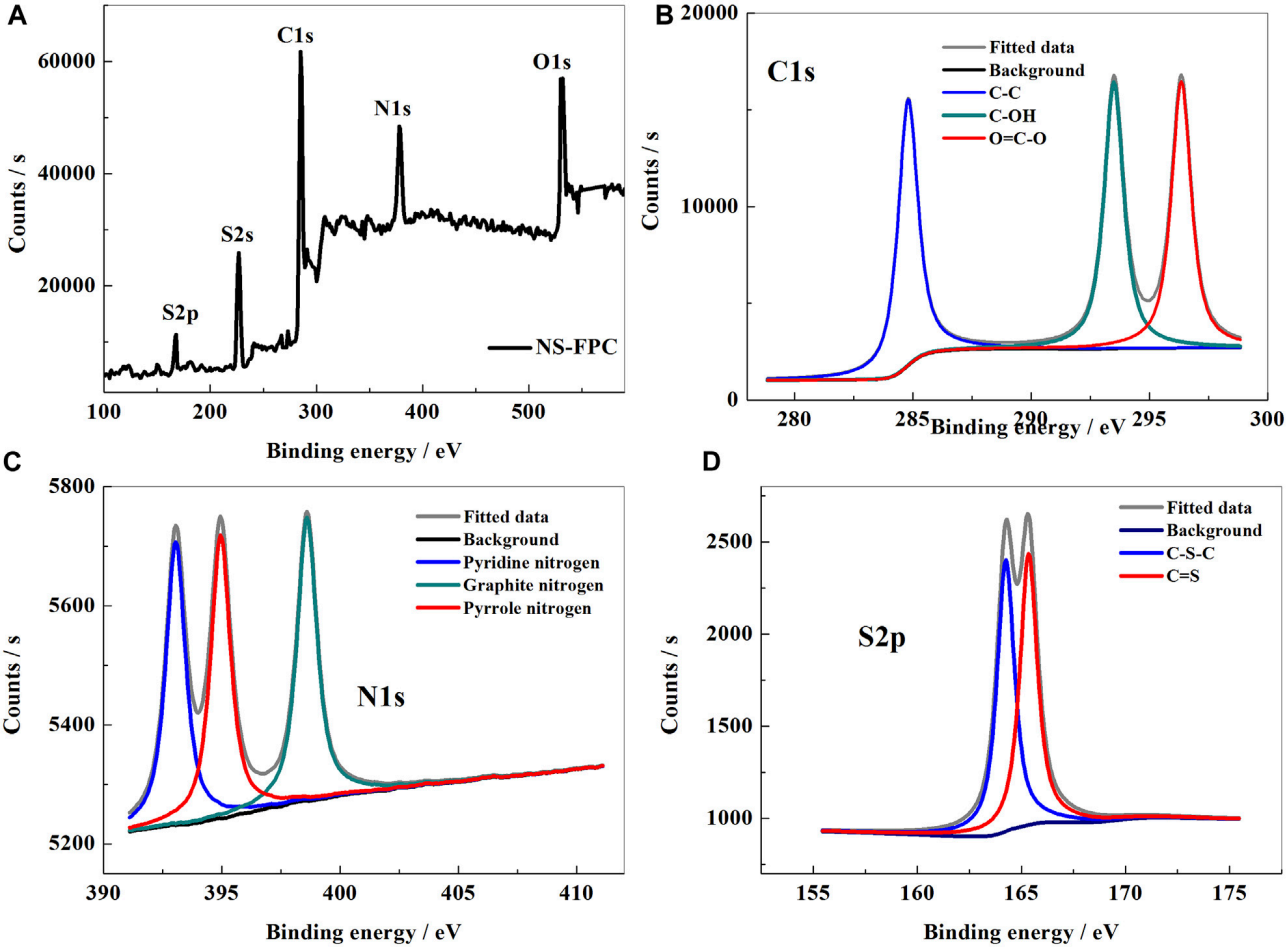
XPS survey **(A)**, Cls **(B)**, N1s **(C)**, and S2p **(D)** spectra of the NS-FPC sample.

In order to explore the existing forms of elements in the NS-FPC matrix, the samples were tested using X-ray photoelectron spectroscopy (XPS), as shown in Figure 2. From the XPS survey in Figure 2A, it can be seen that the NS-FPC matrix exhibits five main characteristic peaks at 532, 396, 285, 227, and 168 eV, attributed to O1s, N1s, C1s, S2s, and S2p peaks, respectively. Among them, the C1s spectra (Figure 2B) can be divided into three independent peaks, corresponding to C-C (284.9 eV), C-OH (294.0 eV), and O=C-O (296.9 eV) (Zhang et al., 2023a; Zhang et al., 2023b). Figure 2C shows the N1s spectral line of NS-FPC and its fitting diagram. As shown in the figure, N1s has three peaks, with the binding energies from low to high being pyridine nitrogen, pyrrole nitrogen, and graphite nitrogen, corresponding to 393.1, 395.0, and 398.6 eV, respectively. Referring to previous literature reports (Chen et al., 2023b; Choi et al., 2023; Niu et al., 2023), pyridine nitrogen can help inhibit the dissolution of lithium polysulfide. It is because the carbon atom doped with pyridine nitrogen is positively charged, while the polysulfide ion is negatively charged. Therefore, nitrogen-doped carbon materials can effectively absorb lithium polysulfide and reduce its dissolution, thereby improving electrochemical performance. From the high-resolution XPS spectra analysis of S2p (Figure 2D), it can be concluded that two characteristic peaks near 164.25 and 165.35 eV correspond to the C-S-C and C=S bonds, respectively. The dual-doping of N–S heteroatoms can effectively improve the conductivity of carbon materials and increase the active site, which is particularly important for enhancing the electrochemical performance of Li–S batteries (Zhao et al., 2022b; Yang et al., 2022; Qi et al., 2023). The XPS results show that nitrogen and sulfur atoms were both successfully doped into the carbon materials.

### 3.3 Morphological analysis of materials

**FIGURE 3:**
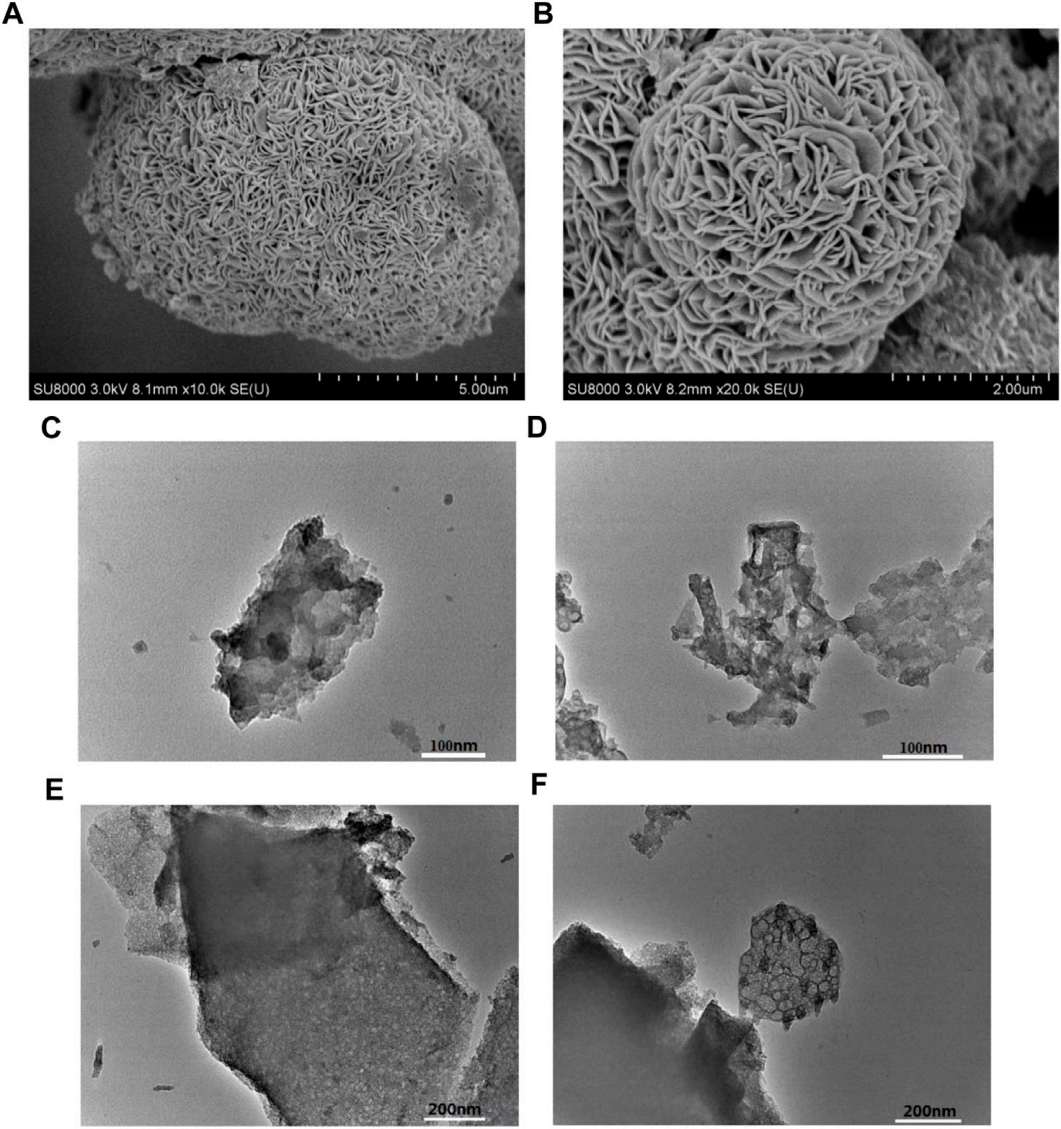
SEM **(A,B)** and TEM **(C–F)** images of the NS-FPC/S sample.

In order to further explore the morphology and structure information of the NS-FPC/S sample, SEM (Figure 3A, B) and TEM (Figures 3C–F) tests were executed. SEM images show that NS-FPC/S is formed by fibrous or scaly aggregates consisting of layered carbon sheets with gaps. This is formed by the removal of ice crystals during freeze-drying. From the TEM results, it can be seen that the NS-FPC/S composites possess an obvious and abundant pore structure, and the pore size is relatively uniform, approximately a few nanometers (micropores and mesopores). As electrode materials for Li–S batteries, these pore structures contribute to the rapid diffusion of ions and reduce electron transfer resistance. The results once again indicate that N and S elements have uniformly dispersed and infiltrated into the matrix of porous carbon.

### 3.4 BET result analysis of materials

**FIGURE 4:**
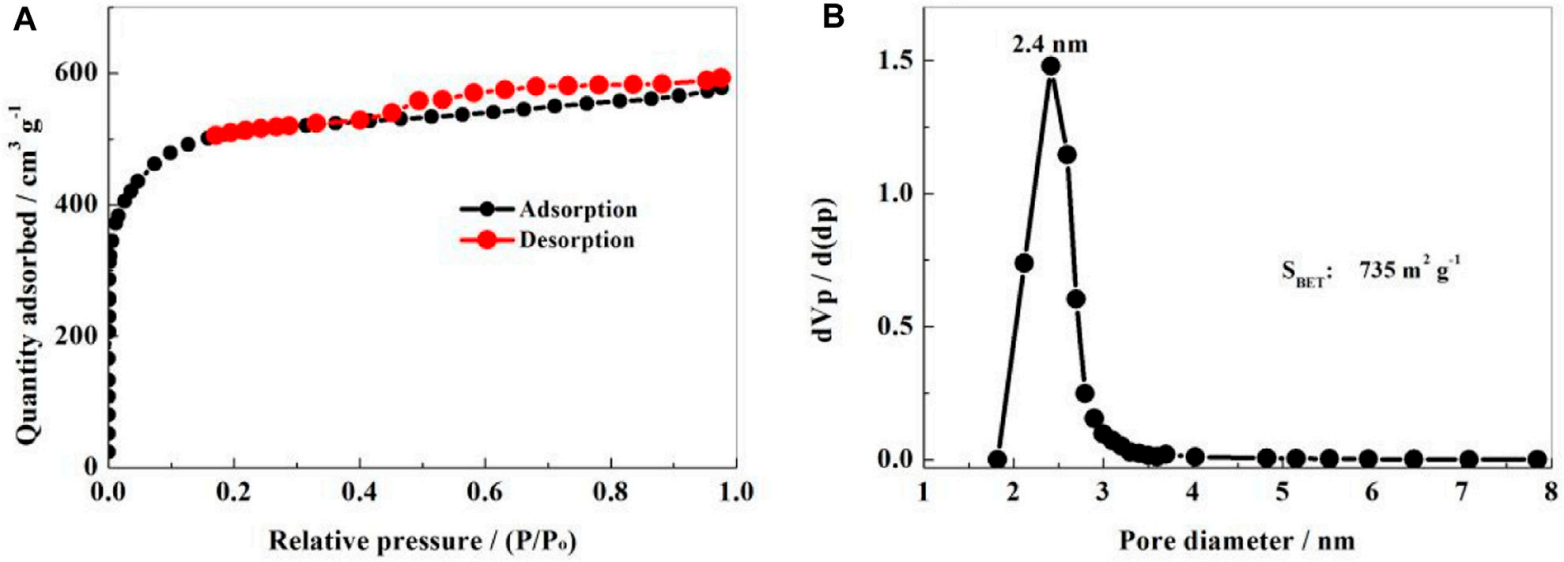
N_2_ adsorption and desorption isotherms **(A)** and pore size distribution curves **(B)** of the NS-FPC sample.

The specific surface area and pore size distribution of the NS-FPC sample were measured through N_2_ adsorption/desorption experiments, as shown in Figure 4. In Figure 4A, the adsorption/desorption equilibrium isotherm can be attributed to a mixture of IUPAC I and IV types. The curve has an obvious adsorption balance platform. The adsorption and desorption curves basically coincide in the area where P/P_0_ is less than 0.42, and an obvious hysteresis loop appears in the area between 0.42 and 1, which is caused by capillary condensation. From Figure 4B, it can be seen that the specific surface area of the material is 735 m^2^ g^-1^, belonging to a mesopore–micropore coexisting structure. The pore size distribution is relatively uniform, concentrating at approximately 2.4 nm, which is consistent with the TEM results. Apparently, the NS-FPC matrix sustains a high specific surface area and uniform porous structure. These porous structures can well accommodate elemental sulfur and alleviate certain volume expansion.

### 3.5 Thermogravimetric result analysis of materials

**FIGURE 5:**
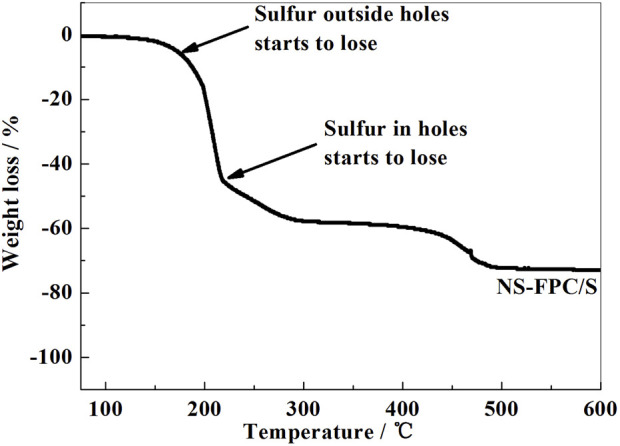
TG curve of the NS-FPC/S sample.

Sulfur was compounded with the prepared NS-FPC matrix using the hot-melt diffusion method, and the weight ratio of sulfur in the NS-FPC/S sample was determined using thermogravimetric analysis. As shown in Figure 5, the curve shows two weight-loss intervals. The range of 155°C–200°C corresponds to the loss of sulfur outside the pores, and the range of 200°C–500°C corresponds to the loss of sulfur inside the pores. Obviously, the thermal stability of sulfur in NS-FPC/S is higher than that of elemental sulfur. This is because of the capillary force of pores in the NS-FPC matrix, which can limit the volatilization of sulfur. From the thermal weight-loss range, it can be calculated that the weight ratio of sulfur in the NS-FPC/S sample is approximately 73%, indicating that the porous structure of the composite material is conducive to the loading of elemental sulfur.

### 3.6 Electrochemical performance analysis of materials

**FIGURE 6:**
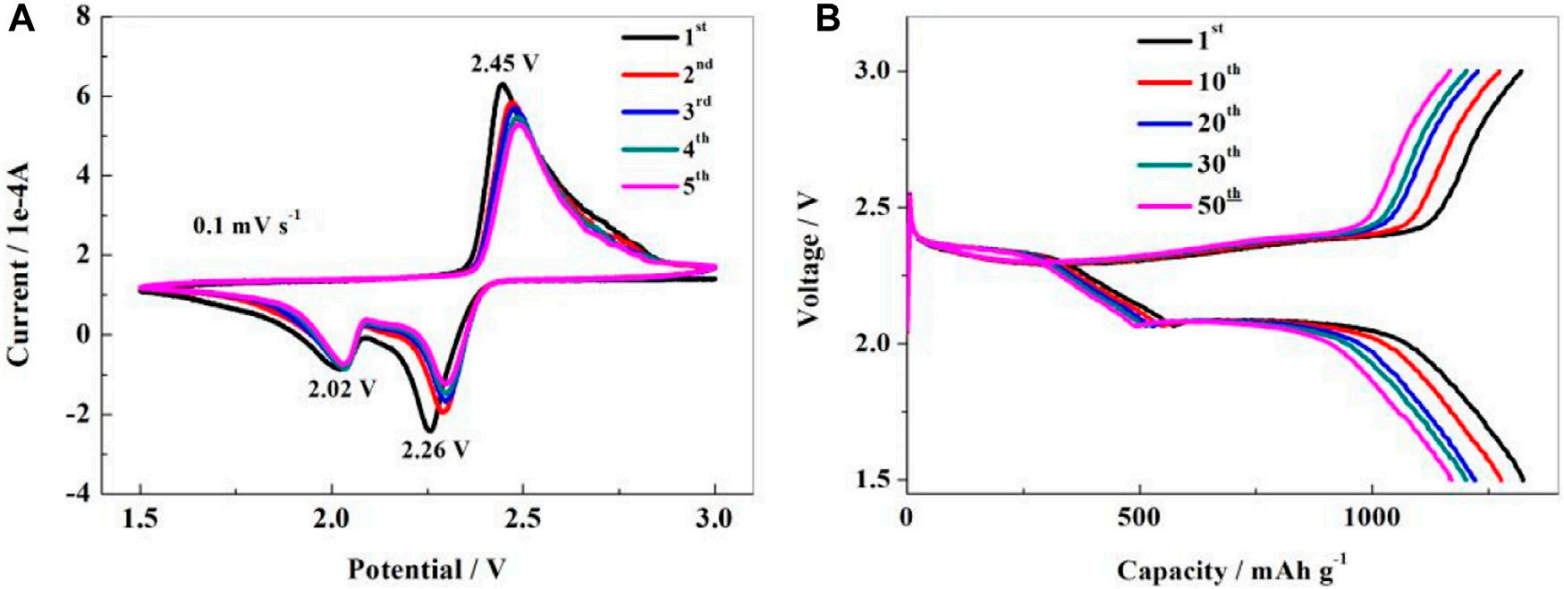
Cyclic voltammograms **(A)** and charge–discharge profiles **(B)** of the NS-FPC/S electrode.

Figure 6A shows the cyclic voltammogram (CV) curve of the NS-FPC/S composite material. It has two obvious reduction peaks at 2.26 and 2.02 V, which correspond to the formation of long-chain lithium polysulfide Li_2_S_8_ intermediate and the reduction process of short-chain lithium polysulfide Li_2_S_x_ (x = 4–6), respectively. When it is reverse-scanned, an oxidation peak appears near 2.45 V, which corresponds to the formation and final oxidation of polysulfide ions to elemental sulfur. In addition, the peak has a narrow shape, and the initial five CV curves can overlap well. This indicates that the NS-FPC/S positive electrode material has relatively good electrochemical reversibility and reaction kinetics. CV results demonstrate that NS-FPC/S composite possesses good conductivity and can effectively inhibit the dissolution of polysulfide. Figure 6B shows the charging–discharging curves of the sulfur positive electrode prepared with NS-FPC as the sulfur storage material at a current density of 0.1°C for the 1st, 10th, 20th, 30th, and 50th cycles. There are two obvious discharge platforms on the discharge curve, which is consistent with the cyclic voltammetry results. The discharge capacity of the NS-FPC/S electrode is relatively high, with a first discharge capacity of 1,324 mAh g^-1^ and a Coulombic efficiency of 98% for the second cycle. This indicates good cycle stability.

**FIGURE 7:**
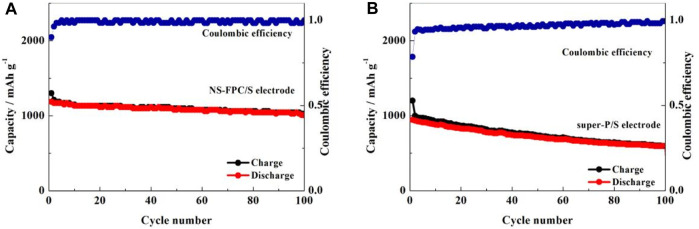
Cycling performance of the sulfur positive electrode based on NS-FPC **(A)** and super-P **(B)** at 0.2°C.

Figure 7 displays the cyclic performance and Coulombic efficiency curves of NS-FPC/S and super-P/S positive electrodes at a current density of 0.2°C. It can be observed that the first discharge capacity of NS-FPC/S decayed from 1,186 mAh g^-1^ to the 100th discharge capacity of 1,008 mAh g^-1^, while the super-P/S decayed from 990 to 559 mAh g^-1^, with capacity retention rates of 85% and 57%, respectively. Through comparison, it can be seen that the cycle stability performance of NS-FPC/S is much better than that of super-P/S. This is mainly due to two aspects. One is that nitrogen–sulfur dual-doping creates a positively charged active site in the matrix material, which can effectively absorb negatively charged polysulfide ions, thus slowing down the dissolution of lithium sulfide in the electrolyte. The second reason is that the porous structure of NS-FPC/S can effectively accommodate sulfur and its compounds and can withstand a certain degree of volume expansion.

**FIGURE 8:**
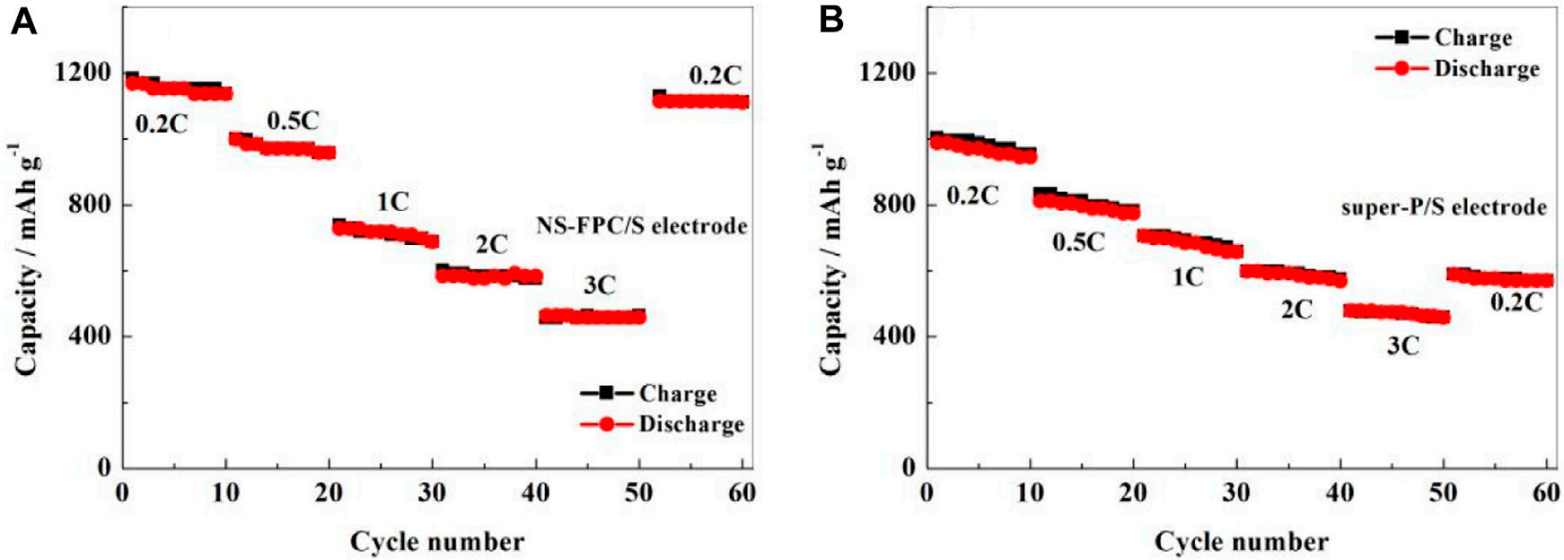
Rate performance of the positive electrode NS-FPC/S **(A)** and super-P/S **(B)**.

The rate performance is also another important indicator for measuring battery performance. Figure 8 shows a comparison of the rate performance using NS-FPC/S and super-P/S as positive electrodes. It can be seen that NS-FPC/S not only has a better cycling performance but also has a significantly better rate performance than super-P/S. When the current density increased from 0.2°C to 0.5 C, 1°C, 2°C, and 3°C and then returned to 0.2°C, the capacities of the NS-FPC/S electrodes were 1,152, 957, 728, 584, 457, and 1,114 mAh g^-1^, respectively. After discharging at different current densities, the current density returned to 0.2°C, and the capacity retention rate reached 96.7%. The NS-FPC/S electrode material can still recover to its capacity at low current after high-current charging–discharging, indicating its superior high-current working ability. Conversely, the super-P/S electrode has a lower capacity at a high current density. When the current density recovered to 0.2°C, its capacity decreased from 987 to 599 mAh g^-1^, with a capacity loss rate of 39.3%.

**FIGURE 9:**
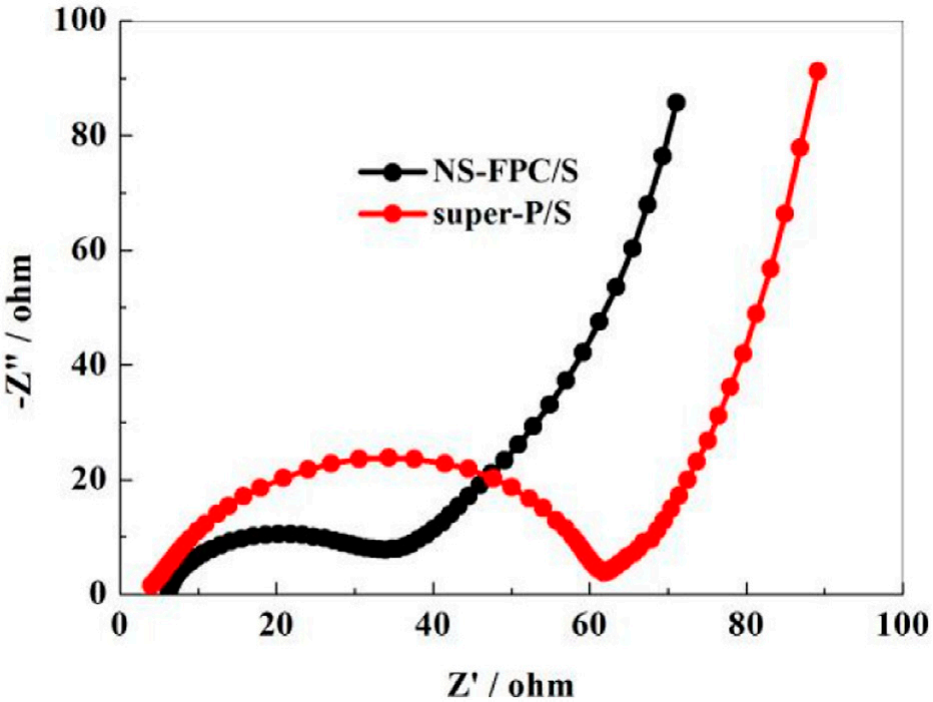
EIS curves of NS-FPC/S and super-P/S electrodes.

Figure 9 shows the EIS curve of NS-FPC/S and super-P/S electrodes before discharge (100 kHz–10 mHz). It consists of two parts: a semicircle in the high-frequency region (left) and a straight line in the low-frequency region (right). The former corresponds to ohmic impedance and charge transfer impedance, while the latter corresponds to Warburg diffusion impedance. The results indicate that the charge transfer resistance and ohmic impedance of NS-FPC/S are both smaller than those of super-P/S. This means that the conductivity of elemental sulfur was significantly increased after combined with the NS-FPC carbon matrix. Surprisingly, compared with previous published articles (Zhao et al., 2019; Zhao et al., 2022a; Zhao et al., 2022b), the electrochemical performance of NS-FPC/S composite showed a considerable improvement.

## 4 Conclusion

In this work, the nitrogen–sulfur dual-doped black fungus porous carbon (NS-FPC) matrix was prepared by hydrothermal, freezing, and calcining processes. The original black fungus was used as the carbon source, cysteine as the nitrogen–sulfur source, and KOH as the activator. This work innovatively achieved the transformation of the original structure of biomass carbon, transforming it from block carbon to porous carbon. The specially constructed form and structure of the materials facilitated activation. The electrochemical performance data show that the NS-FPC/S electrode in Li–S batteries is far superior to the conventionally activated carbon materials. The improvement in electrochemical performance is mainly due to the compounding with the biomass porous carbon matrix and nitrogen–sulfur dual-doping. Compounding with porous carbon increased the conductivity and tolerance to volume expansion. In addition, the positive active site in the carbon materials is caused by nitrogen–sulfur doping, which can effectively absorb the negatively charged polysulfide ions, thus slowing down the dissolution of lithium sulfide in the electrolyte. In a word, the method of preparing a heteroatom-doped porous carbon matrix using natural biological materials in this work has the advantages of simple process and low cost and focuses on green environmental protection. Furthermore, this work has certain environmental protection and commercial application prospects.

## Data Availability

The original contributions presented in the study are included in the article/Supplementary Material, further inquiries can be directed to the corresponding author.
