# Supplementary figures and images for: Preparation of an N–S dual-doped black fungus porous carbon matrix and its application in high-performance Li–S batteries
Source: Front Chem. 2023 Dec 20;11:1288013. doi: 10.3389/fchem.2023.1288013 (PMC10765507; doi:10.3389/fchem.2023.1288013)

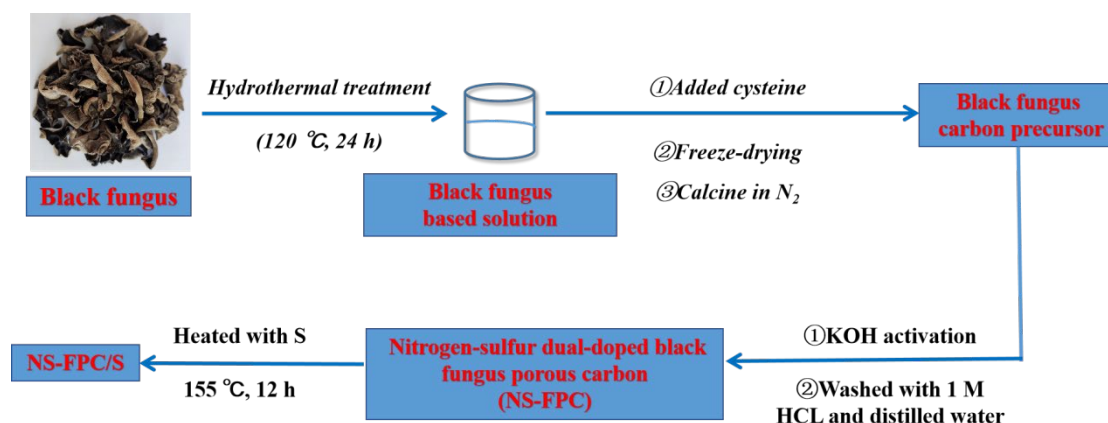

Figure S1. Synthesis process schematic diagram of NS-FPC/S sample.

Supplement: Supplementary file 1 [file Image1.pdf]
